# A Longitudinal Study of Psychosocial and Behavioral Adaptations to Continuous Community Violence in Culiacán, Sinaloa, México

**DOI:** 10.3390/ijerph23081029

**Published:** 2026-08-06

**Authors:** Ángel Radamés Rábago-Monzón, Karyme Mylenia Alemán-Villa, Emiliano Terán, Javier Abednego Magaña-Gómez, Verónica Judith Picos-Cárdenas, Beatriz Kazuko De la Herrán-Arita, Loranda Calderón-Zamora, Alberto Kousuke De la Herrán-Arita

**Affiliations:** 1 Facultad de Medicina, Autonomous University of Sinaloa, Culiacan 80019, Mexico; 2 Facultad de Ciencias Físico-Matemáticas, Autonomous University of Sinaloa, Culiacan 80013, Mexico; 3 Facultad de Ciencias de la Nutrición y Gastronomía, Autonomous University of Sinaloa, Culiacan 80019, Mexico; 4 Unidad Médica de Alta Especialidad, Hospital de Pediatría, Centro Médico Nacional de Occidente, Guadalajara 44340, Mexico; 5 Facultad de Biología, Autonomous University of Sinaloa, Culiacan 80040, Mexico

**Keywords:** Culiacán, perceived insecurity, violence, psychosocial impact, mental health, behavioral adaptation, migration intent, longitudinal study

## Abstract

**Highlights:**

**Public health relevance—How does this work relate to a public health issue?**

- Unmasks the psychological toll of chronic urban warfare: This study demonstrates how prolonged exposure to military-grade cartel conflicts directly spikes community-wide reliance on pharmacologic coping mechanisms and over-the-counter substances to manage acute stress and anxiety.
- Identifies structural barriers to healthy urban mobility: By analyzing neighborhood-level fear, this work links continuous community violence to near-ceiling rates of persistent behavioral restriction, transforming daily safety routines into a permanent lifestyle modification.

**Public health significance—Why is this work of significance to public health?**

- Exposes the “two-speed” nature of trauma adaptation: This research offers rare longitudinal evidence showing that while acute psychological panic and economic shock may quickly subside as populations habituate to danger, defensive behavioral vigilance remains completely rigid.
- Decouples economic hardship from violence-induced displacement: It proves that individual financial strain is not the primary driver of the desire to flee a conflict zone; instead, the profound erosion of community trust and social order serves as the strongest predictor of migration intent.

**Public health implications—What are the key implications or messages for practitioners, policy makers and/or researchers in public health?**

- Demands a shift from individual clinical care to social reconstruction: For practitioners and policymakers, the findings reveal that restoring individual economic stability or providing short-term mental health aid is insufficient to counter the deep, structural damage done to social cohesion and civic trust.
- Reframes behavioral restriction as a primary indicator of population distress: For public health researchers, this study highlights that a decline in acute clinical symptoms does not equal community recovery, proving that long-term tracking must prioritize behavioral mobility over traditional symptom checklists.

**Abstract:**

This study examined how residents of Culiacán, México, adapted over time to prolonged community violence and perceived insecurity. We evaluated a two-speed adaptation theory, which proposes that indicators of acute psychological distress and economic strain decline over time, whereas protective behavioral responses persist as a state of chronic vigilance. A mixed-longitudinal design was employed, consisting of a baseline survey of 1163 residents conducted in October 2024 and a longitudinal cohort of 100 participants followed in February and June 2025. Longitudinal changes were evaluated using Cochran’s Q tests with exact McNemar post hoc comparisons, proportions were reported with 95% Clopper–Pearson confidence intervals, and multivariable logistic regression was used to examine predictors of migration intent. Pharmacologic treatment utilization declined significantly from 36% to 20%, and reported economic strain decreased from 62% to 35% over the study period. In contrast, behavioral routine modifications remained persistently high, decreasing only from 95% to 88%, with no statistically significant longitudinal change. These findings are consistent with the proposed theory, suggesting that while acute psychosocial and economic impacts may diminish over time, protective behavioral adaptations remain comparatively stable. Regarding migration intent, individual economic strain was not significantly associated with the intention to leave the city. Instead, participants who attributed insecurity primarily to social contract collapse, conceptualized as the perceived erosion of community trust, institutional legitimacy, social polarization, and the breakdown of social order, had substantially greater odds of reporting migration intent than those primarily concerned with economic factors (OR = 11.56, 95% CI 1.55–85.98). Overall, these findings suggest that prolonged community violence produces distinct trajectories of psychosocial adaptation, in which acute distress decreases while chronic vigilance persists. Furthermore, migration intentions appear to be more strongly associated with perceived deterioration of the social environment than with individual economic hardship, highlighting the importance of restoring community trust and institutional legitimacy alongside traditional public health and economic interventions.

## 1. Introduction

Chronic community violence represents a profound challenge to urban stability and public health, particularly in regions where organized crime creates a state of “perpetual crisis” [1,2,3,4].

In Culiacán, Sinaloa, México, a city with an estimated population of 1.08 million residents, cycles of high-intensity conflict have moved beyond sporadic events to become a structural feature of daily life. This dynamic was acutely illustrated on 9 September 2024, when an open war erupted within the Sinaloa Cartel (between the Los Chapitos and La Mayiza factions). This conflict led to a wave of armed clashes, burning vehicle blockades (narco-bloqueos), and systemic city-wide paralysis that suspended basic economic and educational activities. While the immediate humanitarian and security costs of such violence are well-documented, the long-term psychosocial and behavioral adaptations of civilian populations in Latin America remain understudied. This is particularly true for Culiacán, which historically serves as a major node of both localized internal displacement and international migration [5,6]. While the immediate humanitarian costs of such violence are well-documented, the long-term psychosocial and behavioral adaptations of the civilian population remain insufficiently understood, often conflating objective exposure to violence with the subjective experience of perceived insecurity [7,8].

### 1.1. Theoretical Framework: Adaptation vs. Vigilance

Previous research examining populations exposed to chronic threat suggests that behavioral avoidance patterns frequently persist after acute emotional responses decline. Such persistence may emerge because avoidance behaviors become reinforced through repeated exposure to perceived danger, ultimately transforming from temporary coping responses into stable components of daily routine. This distinction between emotional adaptation and behavioral persistence provides the conceptual basis for the present investigation.

This study is grounded in the Transactional Model of Stress and Coping proposed by Folkman and Conservation of Resources (COR) theory developed by Hobfoll [9,10]. According to Folkman’s model, individuals continuously judge environmental threats relative to their available coping resources. Under chronic threat, cognitive reappraisal can lead to emotional habituation, where immediate psychological distress and emotional reactions are down-regulated as a survival mechanism. COR theory complements this by asserting that individuals seek to acquire, retain, and foster personal, social, and structural resources. Under chronic danger, individuals engage in a dynamic process of resource allocation, systematically sacrificing secondary resources, such as freedom of movement, leisure time, and social capital, to protect primary resources like physical safety and familial survival.

We propose a “two-speed adaptation” model to reconcile these processes. The Acute Speed (Rapid) is characterized by immediate, sharp spikes in physiological distress, economic panic, and acute coping behaviors. This includes pharmacologic coping behaviors and treatment utilization, which we define as the self-reported use of both prescribed psychiatric medications and self-prescribed or over-the-counter substances to manage acute anxiety. The Chronic Vigilance Phase (Slow Adaptation) is marked by the institutionalization of protective routine modifications [11,12,13]. As acute threats temporarily stabilize, immediate psychological distress and economic panic subside (the acute adaptation phase). However, behavioral modifications, such as changing work commutes, limiting evening travel, or avoiding specific public transit routes, become structurally embedded. This establishes a state of chronic vigilance in which the geography of fear remains permanent despite fluctuations in active gunfire.

### 1.2. From Insecurity to Migration

A critical gap in the current literature is how these internal adaptations (psychosocial and behavioral) translate into the decision to exit the environment entirely. While an extensive body of work in anthropology, sociology, and migration studies has documented forced migration caused by gang violence (such as Maras and MS-13 in Northern Central America) [14,15], less focus has been placed on how structural cartel wars in urban Mexico drive migration intent through non-economic pathways. Migration intent is often framed as a purely economic calculation [16,17]. However, in highly violent contexts, cartel activities inflict severe indirect economic strain, such as forced business closures, extortions, and transportation halts that destroy daily wages. Drawing on theories of the social contract and social trust, we conceptualize social contract collapse as the perceived erosion of institutional protection, community trust, and civic reciprocity under prolonged criminal violence. In this framework, migration in high-risk settings may be driven less by direct financial hardship than by the perception that the institutions and social relationships that normally sustain civic life are no longer able to guarantee safety, predictability, or collective well-being. When non-state actors violently challenge the state, they do not just threaten individuals; they cause social polarization and the decay of community safety nets. When personal vigilance routines and pharmacological coping are deemed insufficient to offset this structural decay, the intent to migrate becomes the ultimate “exit” strategy.

### 1.3. The Present Study

The timing of our data collection provides a unique window into these processes. Spanning from the immediate aftermath of the September–October 2024 escalation of cartel violence through June 2025, when the frequency of large-scale armed confrontations had decreased despite the persistence of chronic insecurity, this longitudinal study tracks a cohort of 100 residents alongside a large-scale baseline (*n* = 1163). We move beyond cross-sectional snapshots to examine the trajectory of three distinct domains: (1) Pharmacologic treatment utilization, (2) Behavioral routine modification, and (3) Economic strain.

We test three primary hypotheses:

**H1.**  *Acute indicators of distress (medication use) and economic shock will show significant longitudinal decline as the population adapts.*

**H2.**  *Behavioral modifications will remain relatively stable, reflecting a persistent state of chronic vigilance, the slow phase of adaptation in which protective behavioral routines become institutionalized even after acute psychological distress begins to decline.*

**H3.**  *High migration intent will be more strongly predicted by perceptions of social polarization and the breakdown of social order than by objective economic strain or demographic factors. Within the framework of H3, “perceived social polarization and the breakdown of social order” is not designed to serve as a proxy for physical violence. Rather, it represents the socio-civic decay, erosion of neighborly trust, and perceived failure of the state-citizen social contract that results from living under the shadow of non-state armed actors. Throughout this manuscript, we use the term social contract collapse as an overarching construct encompassing perceived social polarization, erosion of community trust, weakening of institutional legitimacy, and the breakdown of social order resulting from prolonged organized violence.*

## 2. Methods

### 2.1. Study Design

This study utilized a mixed-longitudinal design. An initial cross-sectional baseline was established in October 2024, from which a longitudinal cohort was derived and followed at two subsequent intervals (February and June 2025).

### 2.2. Sampling and Recruitment

The baseline sample (*n* = 1163) was recruited through a non-probabilistic, voluntary response strategy. Recruitment channels included social media dissemination (Facebook, WhatsApp, X) and in-person outreach at public plazas and community centers in Culiacán. This approach was chosen to maximize reach during a period of active community instability.

The longitudinal cohort (*n* = 100) was selected from a pool of baseline participants who expressed willingness to be re-contacted and provided valid contact information. While demographic comparisons (Table 1) show no significant differences between the baseline and the cohort, we characterize this sub-sample as a convenience-based longitudinal cohort subject to substantial self-selection bias, particularly toward participants with higher civic engagement, perceived insecurity, or interest in the topic. We acknowledge that demographic similarity alone cannot fully exclude attrition bias. Participants who agreed to longitudinal follow-up may differ from non-participants in perceived insecurity, civic engagement, migration intentions, or health-seeking behaviors. Consequently, the longitudinal findings should be interpreted as representative of the retained cohort rather than the entire baseline population. To trace this cohort, our recruitment and retention process was structured as follows: of the 1163 baseline participants who completed the survey in October 2024, a sub-pool of 320 participants expressed explicit interest in longitudinal follow-up and provided validated email addresses or phone numbers. All 320 individuals were re-contacted via email and messaging applications in February 2025 and June 2025. Complete data matching across all three waves was secured for exactly 100 participants, representing a retention rate of 31.3% from the re-contact pool and an overall attrition rate of 68.7% within that sub-group. Unmeasured differences in psychosocial traits may have influenced retention; participants who remained in the study may have possessed higher levels of ongoing security concerns or greater civic commitment than those who dropped out.

### 2.3. Measures and Translation

All survey instruments were originally developed in Spanish. To ensure linguistic and conceptual equivalence for international reporting, items underwent a forward–back translation protocol. Two independent bilingual researchers translated the items into English, and a third researcher back-translated them to Spanish to verify accuracy.

Pharmacologic treatment utilization: Measured by the self-report item: “Have you had to resort to the use of medications (medicamentos) to treat stress or anxiety due to the events of 2024?” This was coded as a binary outcome (Yes/No). We explicitly note that the term “medicamento” in Spanish is frequently interpreted by the local population to include both clinically prescribed psychiatric drugs and self-prescribed, herbal, or over-the-counter remedies. Thus, this variable serves as an indirect proxy of self-reported chemical coping rather than a clinically validated measure of psychiatric prescription severity.

Behavioral routine modification: Assessed by asking if participants modified schedules (work, school, outings). Responses were coded as a binary variable, with “No change” coded as 0 and “Any modification” coded as 1. While practical for sample size limitations, we acknowledge that treating this as a binary variable collapses highly diverse levels of behavioral restriction, ranging from minor route changes to complete self-imposed house arrest, into a single metric, which limits our capacity to measure changes in the intensity of behavioral vigilance.

Economic strain: At baseline, economic impact was measured on a 5-point Likert scale (1 = No impact to 5 = Extreme impact). To ensure alignment with the binary follow-up surveys (which asked “Yes/No” regarding debt accumulation or expense reductions due to violence), baseline scores of 3–5 were recoded into a binary variable (“Yes”), whereas scores of 1–2 were coded as “No”. This practical standardization introduces a potential threat to comparability, and the observed decline from 62% to 35% must be interpreted with caution, as it may partially reflect this structural change in measurement format.

Perceived primary cause of insecurity: Measured using a categorical survey question “What do you perceive as the primary driver of insecurity in your community?” Response options were categorized as: (1) economic and institutional factors, (2) social polarization and community distrust, or (3) active conflict among non-state criminal actors.

Migration intent: Measured on an ordinal scale (Very Probable to Not Probable). For the regression analysis, migration intent was recoded as a binary outcome, with High Intent (Very Probable/Probable) coded as 1 and Low Intent (Not Very Probable/Not Probable) coded as 0 to ensure model stability given the cohort size.

### 2.4. Statistical Analysis

Longitudinal data were analyzed using Cochran’s Q test as an omnibus measure of change across the three waves. Where significant, *post hoc* pairwise comparisons were performed using exact McNemar tests to account for sparse discordant cells in the *n* = 100 cohort. Proportions are reported with 95% Clopper–Pearson binomial confidence intervals.

For Hypothesis 3, a multivariable logistic regression was performed on the June 2025 wave. Given the modest cohort size (*n* = 100) and the number of migration-intent events observed, the regression analysis should be considered exploratory and hypothesis-generating rather than confirmatory. Odds ratios are therefore interpreted primarily in terms of direction and relative magnitude rather than precise effect estimation. The model included the perceived primary cause of insecurity, individual economic strain, and gender as predictors of high migration intent. Baseline associations were tested via Chi-square tests with Cramér’s V reported as a measure of effect size. Missing data were minimal because longitudinal analyses included only participants with complete observations across all three study waves. Therefore, complete-case analysis was performed, and no statistical imputation procedures were required.

## 3. Results

### 3.1. Baseline Characteristics and Cross-Sectional Associations

**Figure 1:**
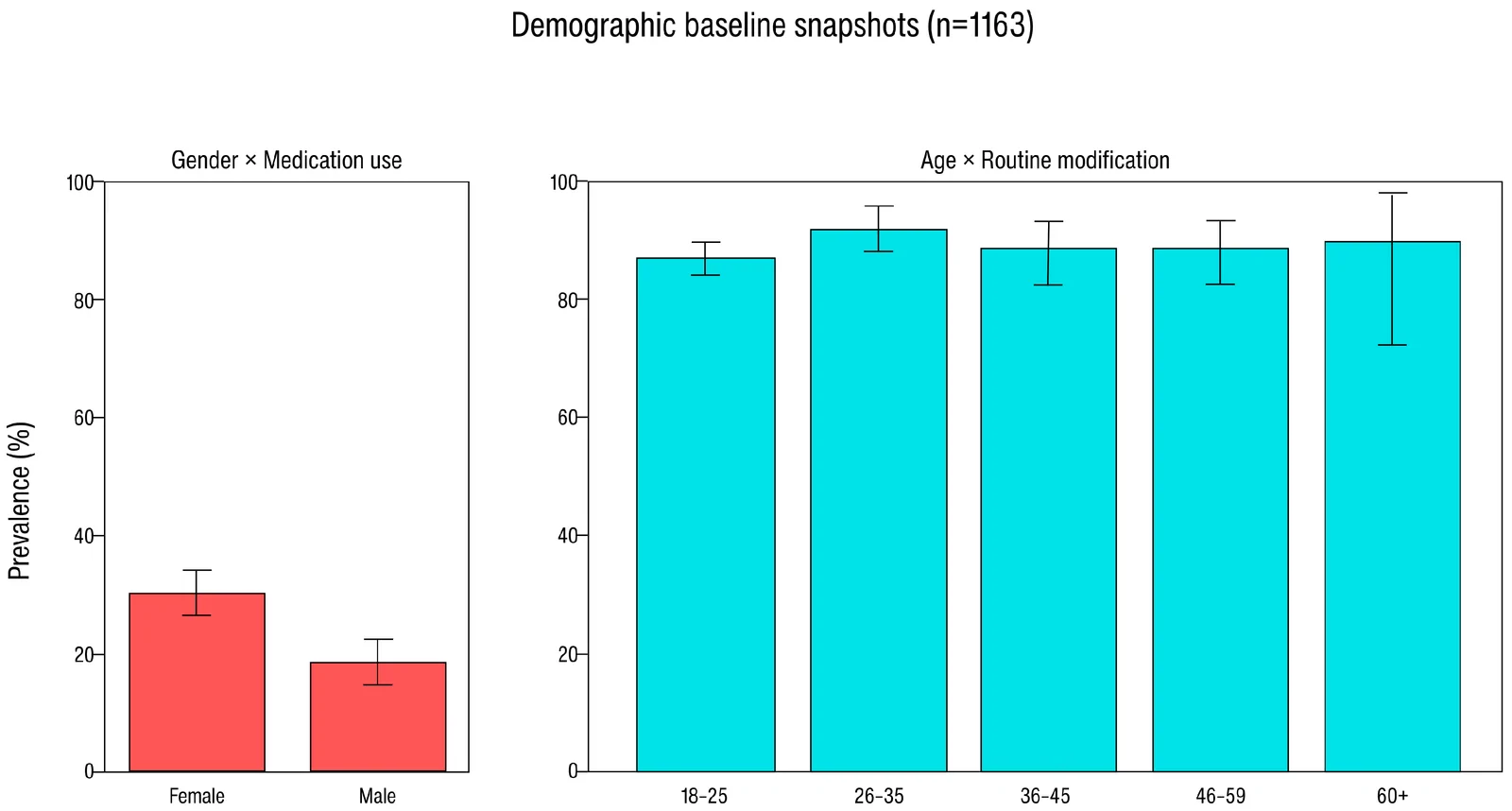
Demographic correlates of baseline impacts (*n* = 1163). Prevalence of psychosocial and behavioral outcomes by demographic groups at the October 2024 baseline. Bars represent the percentage of participants within each demographic category reporting the outcome. Error bars represent 95% binomial confidence intervals. Statistical significance was determined using Pearson’s Chi-square tests (x^2^). Substantive magnitude of associations is reported via Cramér’s V coefficients: gender and pharmacologic treatment (x^2^ = 24.1, *p* < 0.001, V = 0.14); age and routine modification (x^2^ = 18.4, *p* = 0.002, V = 0.11). Note that “Other” gender and “60+” age categories were analyzed using Fisher’s exact tests where cell counts were sparse to ensure inferential stability.

The baseline sample (*n* = 1163) demonstrated widespread impact following the escalation of violence. Significant gender differences were observed in pharmacologic treatment utilization, with women reporting higher usage than men (*p* < 0.001, Cramér’s V = 0.14). Age was significantly associated with routine modifications (*p* = 0.002, Cramér’s V = 0.11), with younger age groups reporting higher rates of behavioral change (Figure 1).

### 3.2. Longitudinal Analysis: The Two-Speed Adaptation

**Figure 2:**
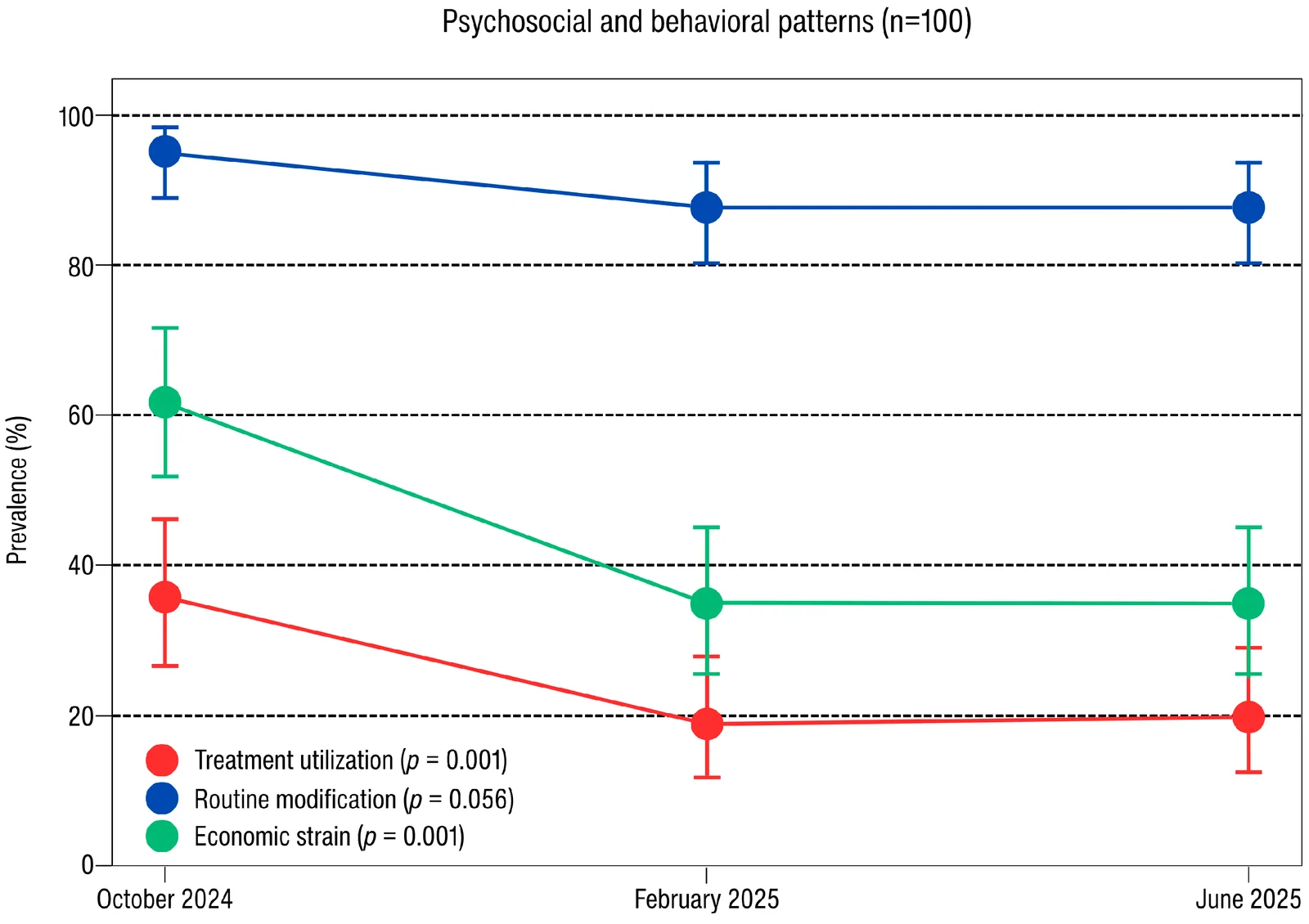
Longitudinal adaptation to chronic insecurity (*n* = 100). Longitudinal trajectories of psychosocial, behavioral, and economic adaptations across three study waves (October 2024, February 2025, and June 2025). Points represent the percentage of the cohort reporting the outcome. Error bars denote 95% Clopper–Pearson binomial confidence intervals. Longitudinal significance across the three waves was assessed using Cochran’s Q omnibus tests. A significant decline was observed in pharmacologic treatment utilization (Q(2) = 15.2, *p* < 0.001) and economic strain (Q(2) = 22.4, *p* < 0.001), while behavioral routine modification remained statistically stable (Q(2) = 5.7, *p* = 0.056), indicating a pattern of chronic vigilance.

Analysis of the longitudinal cohort (*n* = 100) tracked from October 2024 to June 2025 revealed divergent trajectories across psychosocial, economic, and behavioral domains (Figure 2).

Pharmacologic treatment utilization: There was a significant longitudinal decline in the proportion of residents utilizing medication for anxiety/stress, falling from 36% [95% CI: 26.6, 46.2] at baseline to 20% [95% CI: 12.7, 29.2] by June 2025 (Cochran’s Q (2) = 15.2, *p* < 0.001).

Economic strain: Reported economic strain (debt or expense reduction) also showed a marked decrease from 62% [95% CI: 51.7, 71.5] to 35% [95% CI: 25.7, 45.2] over the same period (Cochran’s Q (2) = 22.4, *p* < 0.001).

Behavioral routine modification: In contrast to the decline observed in pharmacologic coping behaviors and reported economic strain, routine modifications remained persistently high. While 95% [95% CI: 88.7, 98.4] of the cohort reported modifications at baseline, 88% [95% CI: 80.0, 93.6] continued these modifications in June 2025. The omnibus Cochran’s Q test indicated no statistically significant longitudinal change (*p* = 0.056), suggesting a state of chronic behavioral vigilance.

Individual trajectory analysis: To transition beyond aggregate-level trends and better evaluate individual-level adaptation trajectories, we conducted a descriptive tracking analysis of the 100 longitudinal cohort members:

Medication vs. routine modification: Out of the 16 individuals who successfully discontinued pharmacologic treatment between October 2024 and June 2025, 14 (87.5%) still maintained active routine modifications in June 2025.

Economic strain vs. routine modification: Of the 27 individuals who reported a reduction in their subjective economic strain over the nine-month period, 24 (88.9%) continued to practice defensive behavioral modifications. These individual trajectories demonstrate that even when acute physical and financial distress indicators de-escalate for a specific resident, their protective vigilance routine remains highly rigid.”

### 3.3. Predictors of Migration Intent

**Figure 3:**
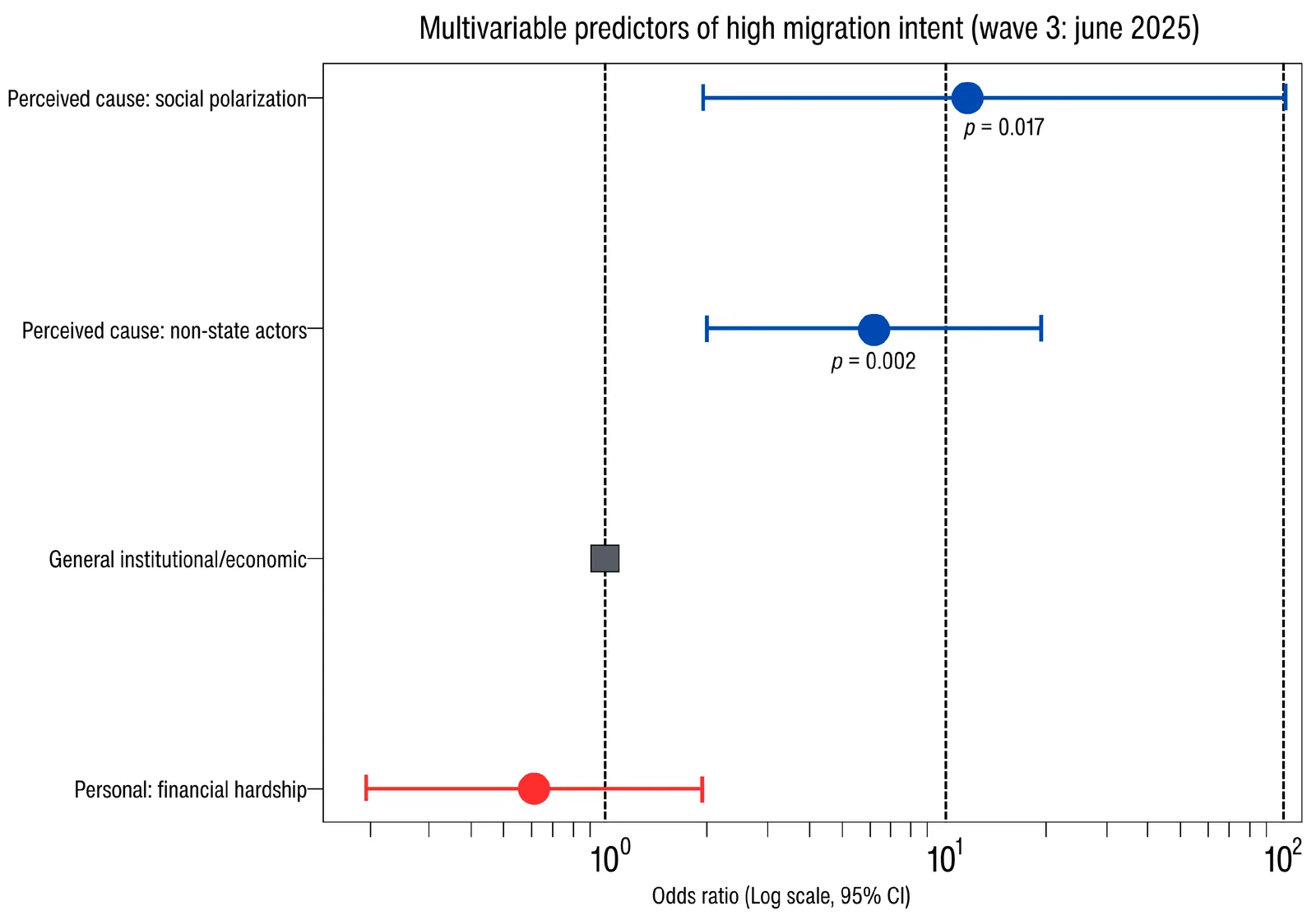
Multivariable predictors of migration intent (June 2025, *n* = 100). Forest plot of odds ratios (OR) for high migration intent based on perceived predictors of insecurity and individual strain. The plot displays ORs and 95% confidence intervals derived from a multivariable logistic regression model. High migration intent (defined as “Probable” or “Very Probable”) served as the dependent variable. The model includes perceived primary causes of insecurity (with “Economic Factors” as the reference category), individual economic strain (binary), and gender (female) as covariates. An OR > 1 indicates increased odds of migration intent. Perceived social polarization demonstrated the strongest statistically significant association with migration intent (OR = 11.56, 95%CI [1.55, 85.9], *p* = 0.017) when controlling for actual reported economic strain (*p* = 0.229).

In the final wave (June 2025), 45% of the cohort reported high migration intent. A multivariable logistic regression was conducted to evaluate the relative impact of perceived insecurity sources and individual economic strain on the desire to leave Culiacán (Figure 3).

The model revealed that perceived social polarization and community distrust was the strongest statistically significant predictor within the multivariable model, although the corresponding confidence interval was wide, indicating substantial uncertainty around the magnitude of the association (OR = 11.56, 95% CI [1.55, 85.98], *p* = 0.017). The wide confidence interval likely reflects the limited sample size and sparse cell counts within certain predictor categories and should therefore be interpreted cautiously. Notably, when controlling for other factors, individual economic strain was not a significant predictor of migration intent (OR = 0.47, 95% CI [0.14, 1.60], *p* = 0.229). Similarly, gender was not a significant predictor in the multivariable context (*p* = 0.893). These findings suggest that the breakdown of the social fabric, rather than direct financial hardship, is the strongest predictor for migration intent in this context.

## 4. Discussion

This longitudinal study provides evidence consistent with a ‘two-speed adaptation’ framework among residents of Culiacán facing chronic community violence. While the observed trajectories support the conceptual model proposed herein, the present data cannot directly establish the existence of distinct underlying adaptation mechanisms. Alternative explanations, including environmental stabilization, changes in healthcare utilization, and behavioral normalization, remain plausible and warrant future investigation. However, our findings suggest that while pharmacologic coping behaviors and reported economic strain declined over time, behavioral vigilance remains remarkably persistent. Furthermore, our analysis reveals that the breakdown of social order, rather than personal economic hardship, is the primary predictor of migration intent in this high-risk urban environment.

### 4.1. The Dynamics of Chronic Vigilance

The observation that 88% of residents maintained routine modifications nine months after the initial escalation (H2), despite a significant decline in medication use (H1), suggests that “adaptation” in a violent context is not synonymous with a return to baseline functioning. Instead, residents appear to enter a state of chronic vigilance [7]. Using the context of Conservation of Resources (COR) theory [6], these behavioral changes represent a protective reallocation of resources. While pharmacologic treatment utilization, used here as an indirect indicator of acute psychological burden, declined over time, persistent perceptions of environmental risk likely continued to shape cognitive appraisal and behavioral decision-making. Rather than representing entirely distinct processes, persistent behavioral restrictions may reflect an enduring psychological representation of threat that continues to influence everyday mobility even after acute emotional distress has partially subsided. In this sense, behavioral vigilance can be interpreted not merely as a behavioral adaptation but also as a sustained cognitive response to chronic insecurity, making it a more stable indicator of perceived risk than acute psychological distress alone.

### 4.2. Social Order and the “Exit” Strategy

A central contribution of this work is the decoupling of economic strain from migration intent (H3). While traditional migration models emphasize “push” factors related to financial survival, our multivariable regression suggests that perceptions of social polarization and community distrust may be more strongly associated with migration intent than direct economic hardship (OR = 11.56). This supports the hypothesis that perceived deterioration in social cohesion and institutional predictability may contribute to migration intent, where migration intent may be influenced by the perception that the state or community can no longer guarantee a predictable social order. In this context, migration is not merely an economic move but a response to the perceived weakening of community cohesion and the rise in non-state actor influence. These findings are broadly consistent with previous research on violence-induced migration in Latin America, which has shown that insecurity, institutional fragility, and the erosion of community trust frequently contribute to displacement decisions alongside, or even beyond, economic considerations. However, whereas much of the existing literature emphasizes direct victimization or objective exposure to violence, our findings suggest that subjective perceptions of social polarization and social order may independently shape migration intentions even after accounting for individual economic strain. This extends previous work by highlighting perceived social contract collapse as a plausible psychosocial pathway linking chronic community violence with migration intent.

### 4.3. Limitations and Alternative Explanations

Several methodological constraints must be considered. First, the observed decline in medication use could partially reflect regression to the mean or changes in healthcare access rather than a purely psychological adaptation. Second, while the longitudinal cohort showed demographic similarity to the large baseline, the *n* = 100 sample is a self-selected group that may overrepresent individuals with higher levels of civic engagement or concern. Future research should utilize probabilistic sampling and validated clinical scales to further distinguish between clinical anxiety and adaptive vigilance. Additionally, pharmacologic treatment utilization should not be interpreted as a direct measure of psychiatric symptom severity. Medication use reflects a complex interaction among symptom burden, healthcare access, physician prescribing practices, treatment adherence, medication costs, perceived stigma, and individual preferences. Consequently, reductions in medication use cannot be assumed to represent equivalent reductions in psychological distress. Similarly, persistent behavioral modification may represent adaptive vigilance, environmental normalization, or constrained mobility behaviors rather than ongoing psychopathology alone.

Furthermore, a key limitation of this study is the complete reliance on perceived, subjective insecurity rather than objective indicators of localized violence. The study period (October 2024 to June 2025) corresponds to the post-crisis stabilization phase of the cartel schism in Culiacán. Because highly localized official crime data (e.g., weekly homicide rates, active blockades, or extortion reports) were unavailable, we cannot definitively determine whether the observed reductions in medication use (36% to 20%) and economic strain (62% to 35%) reflect true psychological habituation or are simply a logical response to a stabilizing security environment. If local violence levels were objectively decreasing during these months, the decline in acute distress would represent environmental tracking rather than internal psychological “adaptation”. We must therefore moderate our causal language, the observed drops in acute markers may reflect a mix of cognitive habituation and external environmental stabilization.

Additionally, although no demographic differences were observed between the baseline sample and retained cohort, unmeasured differences in psychosocial characteristics may have influenced retention. Participants willing to engage in repeated follow-up may have had greater concern regarding insecurity-related issues than non-retained participants.

Finally, all measures relied on self-report, introducing the possibility of recall bias, social desirability bias, and subjective interpretation of survey items. Because perceived insecurity is inherently subjective, participant responses may not perfectly correspond to objective environmental conditions.

## 5. Conclusions

Our findings suggest that populations exposed to chronic violence may not necessarily return to pre-conflict behavioral patterns, even when some indicators of psychosocial and economic distress decline over time. Instead, the observed data are consistent with a process in which behavioral restrictions persist despite reductions in other indicators of distress while maintaining elevated migration intent associated with perceived weakening of social trust. For policymakers, this highlights that restoring economic stability alone may be insufficient to stem migration or improve quality of life; the restoration of the social contract and community trust is paramount.

## Figures and Tables

**Table 1:** Demographic characteristics of baseline sample vs. longitudinal cohort.

Demographic Variable	Baseline Sample (Wave 1) (*n* = 1163)	Longitudinal Cohort (*n* = 100)	Test of Difference (χ^2^ and *p*-Value)
**Gender**			χ^2^ = 0.15, *p* = 0.93 (ns)
Female	58.0%	59.0%	
Male	41.0%	40.0%	
Other	1.0%	1.0%	
**Age group**			χ^2^ = 0.38, *p* = 0.94 (ns)
18–25 years	27.0%	26.0%	
26–35 years	42.0%	46.0%	
36–45 years	20.0%	20.0%	
46–59 years	6.0%	5.0%	
60+ years	5.0%	6.0%	
**Education level**			χ^2^ = 0.53, *p* = 0.91 (ns)
Basic education	25.0%	24.0%	
High school	40.0%	41.0%	
University	30.0%	29.0%	
Postgraduate	5.0%	6.0%	

Note: ns, not significant.

## Data Availability

The datasets generated and analyzed during the current study are not publicly available, but are available from the corresponding author on request, provided appropriate ethical and data usage agreements are in place.
